# Structural basis of Cas3 activation in type I-C CRISPR-Cas system

**DOI:** 10.1093/nar/gkae723

**Published:** 2024-08-24

**Authors:** Do Yeon Kim, So Yeon Lee, Hyun Ji Ha, Hyun Ho Park

**Affiliations:** College of Pharmacy, Chung-Ang University, Seoul 06974, Republic of Korea; Department of Global Innovative Drugs, Graduate School of Chung-Ang University, Seoul 06974, Republic of Korea; College of Pharmacy, Chung-Ang University, Seoul 06974, Republic of Korea; Department of Global Innovative Drugs, Graduate School of Chung-Ang University, Seoul 06974, Republic of Korea; College of Pharmacy, Chung-Ang University, Seoul 06974, Republic of Korea; College of Pharmacy, Chung-Ang University, Seoul 06974, Republic of Korea; Department of Global Innovative Drugs, Graduate School of Chung-Ang University, Seoul 06974, Republic of Korea

## Abstract

CRISPR-Cas systems function as adaptive immune mechanisms in bacteria and archaea and offer protection against phages and other mobile genetic elements. Among many types of CRISPR-Cas systems, Type I CRISPR-Cas systems are most abundant, with target interference depending on a multi-subunit, RNA-guided complex known as Cascade that recruits a transacting helicase nuclease, Cas3, to degrade the target. While structural studies on several other types of Cas3 have been conducted long ago, it was only recently that the structural study of Type I-C Cas3 in complex with Cascade was revealed, shedding light on how Cas3 achieve its activity in the Cascade complex. In the present study, we elucidated the first structure of standalone Type I-C Cas3 from *Neisseria lactamica* (NlaCas3). Structural analysis revealed that the histidine–aspartate (HD) nuclease active site of NlaCas3 was bound to two Fe^2+^ ions that inhibited its activity. Moreover, NlaCas3 could cleave both single-stranded and double-stranded DNA in the presence of Ni^2+^ or Co^2+^, showing the highest activity in the presence of both Ni^2+^ and Mg^2+^ ions. By comparing the structural studies of various Cas3 proteins, we determined that our NlaCas3 stays in an inactive conformation, allowing us to understand the structural changes associated with its activation and their implication.

## Graphical Abstract

**Graphical Abstract:**
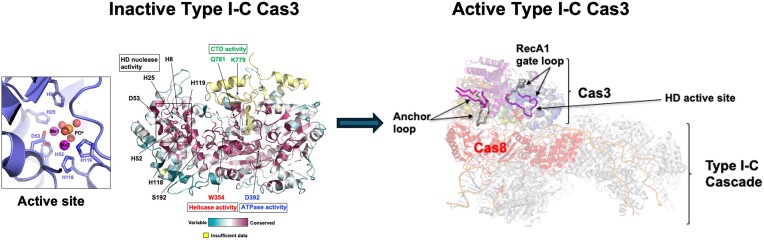

## Introduction

Bacteria have undergone myriad evolutionary adaptations to overcome the threats originating from the perpetual warfare between bacteria and their assailants, such as bacteriophages and various mobile genetic elements (1). The emergence of clustered regularly interspaced short palindromic repeats (CRISPRs) and their associated proteins (Cas) signifies a pivotal milestone in bacterial defense strategies, endowing bacteria with immunity against intrusive genetic materials (2–5). CRISPR–Cas systems possess an adaptive trait, whereby they meticulously archive memories of past infections within their CRISPR arrays, thereby orchestrating a swift immune response upon subsequent encounters (6).

The functionality of CRISPR–Cas systems unfold through a triadic sequence of events including adaptation, expression, and interference (7,8). During the adaptation phase, infiltrating DNA is processed and assimilated as spacers into the CRISPR array nested within the bacterial genome (9). During the expression phase, the host CRISPR array undergoes transcription and processing, resulting in diminutive CRISPR RNAs (crRNAs). Finally, in the interference phase, complexes guided by crRNAs recognize sequences complementary to the crRNA in invaders, executing cleavage either autonomously or by enlisting additional proteins to dismantle DNA or RNA of the invaders (8,10,11). CRISPR–Cas systems have been harnessed for gene editing owing to their remarkable precision in target DNA cleavage, with ongoing trials exploring their potential in treating various diseases (12–16).

The long evolutionary interaction between bacteria and phages has led to the diversification of CRISPR–Cas systems that are currently grouped into two broad classes (classes 1 and 2) encompassing six types (types I–VI) based on the organization of CRISPR locus, composition of *cas* and their mechanisms (17). The class 1 systems, including types I, III and IV, employ multi-subunit Cas proteins for performing multiple functions, whereas the class 2 systems, including types II, V and VI, utilize a single multi-domain Cas that performs all necessary activities (17).

The Type I CRISPR-Cas systems are the predominant and extensively dispersed class, delineated into seven distinct subtypes, denoted as I-A through I-G, and contingent upon their distinctive *cas* genes and composition of the Cas constituents (17). Within the Type I systems, numerous Cas proteins orchestrate collaborative work with crRNA for amalgamating and forming a formidable CRISPR-associated complex for antiviral defense (Cascade), which recognizes invader DNA and finally recruits Cas3, the nuclease responsible for destroying target DNA (11).

Cas3 is a multi-domain protein consisting of approximately 800–900 amino acids in the Type I CRISPR–Cas system and is responsible for the actual cleavage of target DNA (18). Upon recognition of foreign DNA via the Cascade, Cas3 is recruited to the site and initiates DNA degradation. The enzymatic activity of Cas3 results in irreversible destruction of the target DNA, thereby effectively neutralizing the threat posed by invading genetic materials (19). Precise coordination between Cas3 and other components of the CRISPR–Cas system ensures the specificity and efficiency of immune response, allowing bacteria to fend off viral attacks and maintain genomic integrity. The structures of Cas3 proteins acting in some Type I CRISPR–Cas systems were elucidated a long time ago (20–23). However, the structural study of Type I-C Cas3 in the Cascade complex was only revealed recently, providing insights into how Cas3 functions within the Cascade complex (24). Nonetheless, for Type I-C Cas3, there is still a lack of structural studies on its inactive form when it exists alone before becoming active within the Cascade complex. This gap in knowledge prevents a complete understanding of the full mechanism of Cas3 activation and inactivation.

In the present study, we biochemically characterized and determined the first structure of standalone Type I-C Cas3 from *Neisseria lactamica* (hereafter referred to as NlaCas3) for understanding the molecular basis underlying the activation and inactivation of Cas3 in the Type I-C CRISPR–Cas system. Because our standalone structure of NlaCas3 was able to represent the inactive state of Type I-C Cas3, and based on structural and biochemical studies along with the recently published cryogenic electron microscopic (Cryo-EM) structure of the Type I-C Cascade/Cas3 complex, we demonstrate the structural basis of activation and inactivation mechanism of Cas3 in the Type I-C CRISPR–Cas system.

## Materials and methods

### Cloning, overexpression, and purification of Cas3 for structural and biochemical studies

Full-length *cas3* (encoding residues 1–834) of *N. lactamica* (GenBank: WP_036469581.1) was purchased from Addgene (plasmid #180214). The resulting recombinant construct was transformed into *Escherichia coli* BL21(DE3) competent cells, which were subsequently grown at 37°C in 1 l lysogeny broth containing 50 μg/ml kanamycin. When the optical density at 600 nm reached approximately 0.7–0.8, temperature was adjusted to 20°C, and 0.5 mM isopropyl β-d-1-thiogalactopyranoside was added to induce Cas3 expression. Induced cells were further cultured for 16 h in a shaking incubator at 20°C. Cultured cells were harvested by centrifugation at 2000 × *g* for 15 min at 4°C, resuspended in 20 ml lysis buffer (20 mM Tris–HCl pH 8.0 and 500 mM NaCl), and lysed by ultrasonication. The cell lysate and supernatant were separated by centrifugation at 10 000 × *g* for 30 min at 4°C. The supernatant was combined with Ni-nitrilotriacetic acid (NTA) affinity resin for 2 h and loaded onto a gravity-flow column (Bio-Rad, Hercules, CA, USA). The resin in the column was washed with 25 ml wash buffer (20 mM Tris–HCl pH 8.0 and 25 mM imidazole) to remove unbound proteins. Following the washing step, 3.25 ml elution buffer (20 mM Tris–HCl, pH 8.0, 500 mM NaCl, and 250 mM imidazole) was added to the column to elute the target protein bound to Ni-NTA resin.

Eluted Cas3 was concentrated to 30 mg/ml and applied onto a Superdex 200 10/300 GL column (GE Healthcare, Waukesha, WI, USA) connected to an ÄKTA Explorer system (GE Healthcare). The column was preequilibrated with size-exclusion chromatography (SEC) buffer (20 mM Tris–HCl pH 8.0 and 150 mM NaCl) for polishing protein sample by SEC. The eluted peak fractions containing Cas3 were collected, pooled, and concentrated to 3.3–3.5 mg/ml for crystallization. Protein purity was visually assessed by sodium dodecyl sulfate–polyacrylamide gel electrophoresis (SDS-PAGE).

### Crystallization and X-ray diffraction

The sitting drop vapor diffusion method was employed for crystallizing Cas3. Crystal plates were incubated at 20°C. Initial crystals formed by equilibrating a mixture of 0.3 μl protein solution (3.3 mg/ml in SEC buffer) and 0.3 μl reservoir solution containing 10% (w/v) polyethylene glycol 8000, 0.1 M sodium/potassium phosphate at pH 6.2, and 0.2 M NaCl, equilibrated against 80 μl of the reservoir solution. The crystallization conditions were further optimized, and the best crystals were obtained by increasing pH of sodium/potassium phosphate buffer to 6.6–6.8. For X-ray diffraction experiments, a single crystal was selected and soaked in reservoir solution supplemented with 40% (v/v) glycerol for cryoprotection. X-ray diffraction data were collected at −178°C on the beamline BL-5C at the Pohang Accelerator Laboratory (Republic of Korea). The data were processed using HKL2000 software (25).

### Structure determination and refinement

Cas3 structure was determined by the molecular replacement phasing method using PHASER program in the PHENIX package (26). The predicted structural model generated by AlphaFold2 was used as a search model (27). The initial model was automatically constructed using AutoBuild in the PHENIX package, with subsequent model building and refinement using Coot (28) and phenix.refine (29). The structural quality and stereochemistry were validated using MolProbity (30). All structures were generated using PyMOL (31).

### Multi-angle light scattering analysis (MALS)

The absolute molecular weight of Cas3 in solution was determined utilizing size exclusion chromatography coupled with multi-angle light scattering (SEC-MALS). Protein solution was loaded onto a Superdex 200 Increase 10/300 GL 24-ml column (GE Healthcare) preequilibrated with SEC buffer. The flow rate was maintained at 0.4 ml/min, and SEC-MALS analysis was conducted at 20°C. A DAWN-TREOS MALS detector was linked to an ÄKTA Explorer system. The molecular weight of bovine serum albumin was used as the reference value. Data processing and evaluation were carried out using ASTRA.

### Assay of cleavage activity of Cas3 *in vitro*

Target DNA oligonucleotides containing the identified spacer sequences were synthesized by Bionics (Daejeon, Republic of Korea) (Supplementary Table S1). Two complementary single-stranded DNAs (ssDNA) were incubated at 100°C for 3 min and then slowly cooled down to room temperature for generating double-stranded DNA (dsDNA).

A series of *in vitro* target DNA cleavage assays were conducted for testing the nuclease activity of wild-type Cas3 and its mutants (H25A, H52A, D53A, H118F, H119A, S192A, D392A, W354A, K779A, and Q781A). Reactions were performed in 10 μl nuclease buffer (40 mM Tris–HCl pH 8.0, 6 mM MgCl_2_, 0.4 mM Ni(II)SO_4_, 8 mM ATP, 0.4 mg/ml and bovine serum albumin) by adding 5 μl of either wild-type (5–400 nM) or mutant (20–50 nM) Cas3 and 5 μl of 42-bp Cas3 target dsDNA or ssDNA (30 nM). The reaction mixture was incubated at 37°C for 2 h. The reaction was terminated by adding 10 μl stop solution [67.5 mM ethylenediaminetetraacetic acid (EDTA), 27% (v/v) glycerol, 0.3% (w/v) SDS] and incubating for 10 min at room temperature. Reactions were conducted with or without 8 mM ATP to assess it role on DNA cleavage.

For ion-dependent cleavage assay, the same procedure was followed, except for the nuclease buffer. A buffer was prepared with all ions removed, and then the following ions at specific concentrations were added: 6 mM MgCl_2_, 0.4 mM NiSO_4_, 0.4 mM CoCl_2_, 0.4 mM FeCl_2_ and 0.4 mM MnCl_2_. All samples were then separated by electrophoresis at 100 V on a 14% native 0.5 × Tris–borate–EDTA polyacrylamide gel for 90 min. After electrophoresis, gels were stained with SYBR Gold (Invitrogen, Waltham, MA, USA) and visualized according to the manufacturer's instructions. Relative amounts of DNA in the gel were quantified using ImageJ, a public domain Java-based image processing program.

### Inductively coupled plasma-mass spectrometry (ICP-MS) analysis

The concentration of trace metal ions in NlaCas3 was determined through a comparative analysis involving a serial dilution of the Recipe control samples (Munich, Germany) prepared in water. To ensure accuracy, a combination of Be and Co internal standards was introduced into both calibration points and samples at specific concentrations. Measurements were conducted using a NexION350D ICP–MS (SCIEX model; Perkin-Elmer) equipped with an argon plasma source. The analysis was conducted at the National Center for Inter-University Facilities at Seoul National University, Seoul, Republic of Korea. Sample was introduced at a rate of 1.00 ml/min during measurement. The data represent the average values derived from triplicate samples, ensuring a robust assessment of the concentrations of trace metal ions in Cas3.

### Sequence alignment

The amino acid sequences of Cas3 across different species were analyzed using Geneious Prime v.2024.0.3 (https://www.geneious.com).

### Mutagenesis

Site-directed mutagenesis was performed using a QuickChange kit (Stratagene, San Diego, CA, USA) according to the manufacturer's protocol. Mutations were confirmed by sequencing. Mutant proteins were prepared using the same method described above for purification of wild-type protein.

### Electrophoretic mobility shift assay (EMSA)

Varying concentrations of purified CTD domain of NlaCas3 were pre-incubated with 30 nM of double strand DNA or single strand DNA in binding buffer (10 mM HEPES pH 7.5, 1 mM MgCl_2_ 20 mM KCl, 1 mM tris(2-carboxyethyl)phosphine (TCEP), and 5% (v/v) glycerol in a final volume of 20 μl) for 30 min on ice. Prepared samples were then separated by gel electrophoresis at 100 V on a 10% native 0.5× TBE (Tris borate EDTA) polyacrylamide gel. After electrophoresis, gels were stained with SYBR Gold (Invitrogen, Waltham, MA, USA) and visualized according to the manufacturer's instructions.

## Results

### Biochemical characterization and determination of the structure of type I-C Cas3 from a *N. lactamica*

**Figure 1.**
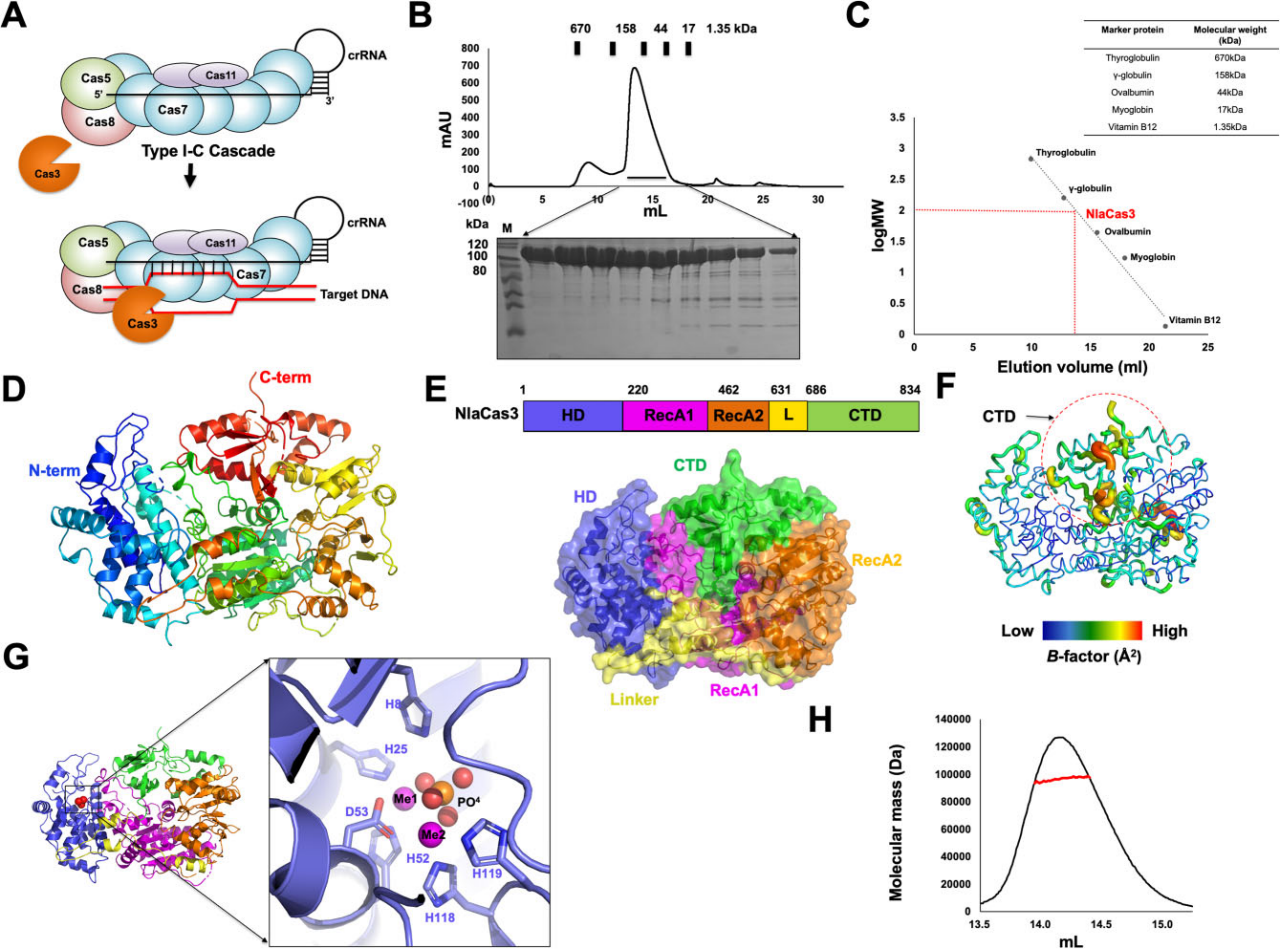
The crystal structure of Type I-C Cas3 from *N. lactamica*. (**A**) An overview of the assembly and target degradation process of the Type I-C CRISPR-Cas system. (**B**) SEC profile of NlaCas3. The picture of a gel after SDS–PAGE, loaded with the peak fractions is provided under the profile. Loaded fractions are indicated by the horizontal black bar. The corresponding fractions from SEC loaded on the gel are indicated by black arrows. M indicates protein marker. (**C**) The elution volume is fitted in the SEC vs. the size marker and log of molecular weight of NlaCas3. A red point on the fitting line marks the elution volume. Molecular weights of the size markers are compiled in the table. (**D**) The overall structure of NlaCas3. Cartoon representation of NlaCas3. Color of the chain from the N- to the C-terminus gradually moves through the spectrum from blue to red. (**E**) Domain composition and boundary of NlaCas3. (**F**) *B*-factor distribution in NlaCas3 structure. The structure is shown by a putty representation. Rainbow colors from red to violet with increasing values were used for *B-*factor visualization. The region with the highest B-factor, corresponding to most of the CTD, is indicated by a red-dot circle. (**G**) Position of the metal ion-binding site and a close-up view of the active site of the HD nuclease domain of NlaCas3, to which the metal ions are bound. The metal-coordinating residues are labelled. (**H**) MALS profile of NlaCas3. The experimental MALS data (red line) are plotted as SEC elution volume (x-axis) versus absolute molecular mass (y-axis) distributions on the SEC chromatogram (black) at 280 nm.

The Type I CRISPR-Cas system is a subtype within the broader CRISPR-Cas system family, characterized by its unique features and functionalities. This type utilizes a multi-protein complex, known as Cascade, guided by RNA, to locate specific DNA targets for cleavage. Once the Cascade complex identifies the target DNA, Cas3, which possesses both nuclease and helicase activities, is recruited to degrade the target DNA (Figure 1A). Among the seven subtypes, Type I-C stands out for its compactness and remarkable efficacy in inducing substantial genome editing (19,32). In the Type I-C CRISPR-Cas system, several subunits of Cas proteins, such as Cas5, Cas7, Cas8 and Cas11, come together to construct an RNA-guided multi-subunit Cascade complex (32,33).

Although the structures of standalone Cas3 proteins involved in certain Type I CRISPR-Cas systems have been unveiled, the structural intricacies of standalone Type I-C Cas3 have remained elusive. To unravel the molecular fundamentals governing the activation and inactivation of Cas3 in the Type I-C CRISPR-Cas system, we conducted a structural study of standalone NlaCas3. For structural analysis, NlaCas3 was overexpressed in *E. coli* and purified using affinity chromatography followed by SEC. SEC analysis revealed that NlaCas3 exists as a monomer in solution, as evidenced by an elution peak at approximately 14 ml, falling between the elution volumes of γ-globulin (158 kDa) and ovalbumin (44 kDa) (Figure 1B and C). Following purification, the target protein was successfully crystallized, and its crystal structure was determined at a resolution of 2.17 Å using the molecular replacement (MR) phasing method. The predicted structure generated by AlphaFold2 was utilized as the search model during this process (27). The final structural model of NlaCas3 was refined, yielding *R*_work_ = 19.22%, and *R*_free_ = 24.96%. The detailed diffraction data and refinement statistics are presented in Table 1.

**Table 1.** Data collection and refinement statistics

Data collection	
Space group	*C 1 2 1*
Unit cell parameter *a*, *b*, *c* (Å)	
*a*, *b*, *c* (Å)	*a* = 124.97, *b* = 73.33, *c* = 104.42
*α*, *β*, *γ* (°)	*α* = 90, *β* = 99.749, *γ* = 90
Resolution range (Å)^a^	29.42–2.17
Total reflections	340 229
Unique reflections	49 373
Multiplicity	6.9 (7.1)
Completeness (%)^a^	99.89 (99.88)
Mean *I*/σ(*I*)^a^	12.49 (1.94)
*R*_merge_ (%)^a,b^	10.49 (0.9672)
Wilson *B*-factor (Å^2^)	36.54
**Refinement**	
Resolution range (Å)	29.42–2.17
Reflections	49 373
*R*_work_ (%)	19.22 (26.28)
*R*_free_ (%)	24.96 (32.78)
No. of molecules in the asymmetric unit	1
No. of nonhydrogen atoms	6570
Macromolecules	6229
Solvent	339
Average *B*-factor values (Å^2^)	44.76
Macromolecules	44.69
Solvent	46.03
Ramachandran plot:	
favored/allowed/outliers (%)	97.78 / 2.22 / 0.00
Rotamer outliers (%)	1.69
Clashscore	6.14
RMSD bonds (Å) / angles (°)	0.008 / 0.94

^a^Values for the outermost resolution shell in parentheses.

^b^R_merge_ =Σ_h_ Σ_i_ |*I*(*h*)_i_ − <*I*(*h*)>|/ Σ*_h_* Σ*_i_* I(*h*)*_i_*, where *I*(*h*) is the observed intensity of reflection h, and < *I*(*h*)> is the average intensity obtained from multiple measurements.

The crystal was categorized under the space group *C2*, with a single molecule present in the asymmetric unit. The final structural model encompassed most of the NlaCas3 sequence, spanning residues D4–D821. The three N-terminal and 13 C-terminal residues were not included in the final structural model because of their invisible electron densities.

The overall tertiary structure of NlaCas3 exhibited a typical structural fold of Cas3, compactly arranged with the N-terminal histidine–aspartate (HD) nuclease domain and RecA-like helicase domains positioned adjacent to each other, thereby forming a functional unit that is capable of DNA cleavage and unwinding (Figure 1D and E). The C-terminal DNA-binding domain (CTD) extended outward, enabling interactions with the target DNA. *B*-factor analysis showed that most of the CTD had relatively higher *B*-factors (average 48.25 Å^2^) than those of the most other parts (average 61.9 Å^2^), indicating that the CTD of NlaCas3 might be flexible in solution (Figure 1F). Metal densities corresponding to the two metal ions in the active site of HD nuclease of NlaCas3 were identified. Additionally, PO4 density around the metal ion was noticed. These two metals and one PO4 were coordinated by five surrounding histidine residues (H8, H25, H52, H118 and H119) and one aspartic acid residue (D53) (Figure 1G).

While most Cas proteins within the CRISPR–Cas system typically function as monomers, certain Cas proteins, such as Cas7, form oligomers that perform their roles within the system. Therefore, we employed MALS to validate the stoichiometry of NlaCas3 by determining its absolute molecular mass in solution. MALS analysis revealed an experimental molecular mass of 96.5 kDa with a fitting error of 7.1% and polydispersity of 1.002 (Figure 1H). Considering that the theoretically calculated molecular weight of monomeric NlaCas3 with a C-terminal histidine tag is 100.5 kDa, the molecular mass determined by MALS likely corresponded to that of monomeric NlaCas3. Based on these SEC and MALS findings, we concluded that NlaCas3 exists as a monomer in solution.

### NlaCas3 can cleave both dsDNA and ssDNA in a metal ion-dependent manner

**Figure 2.**
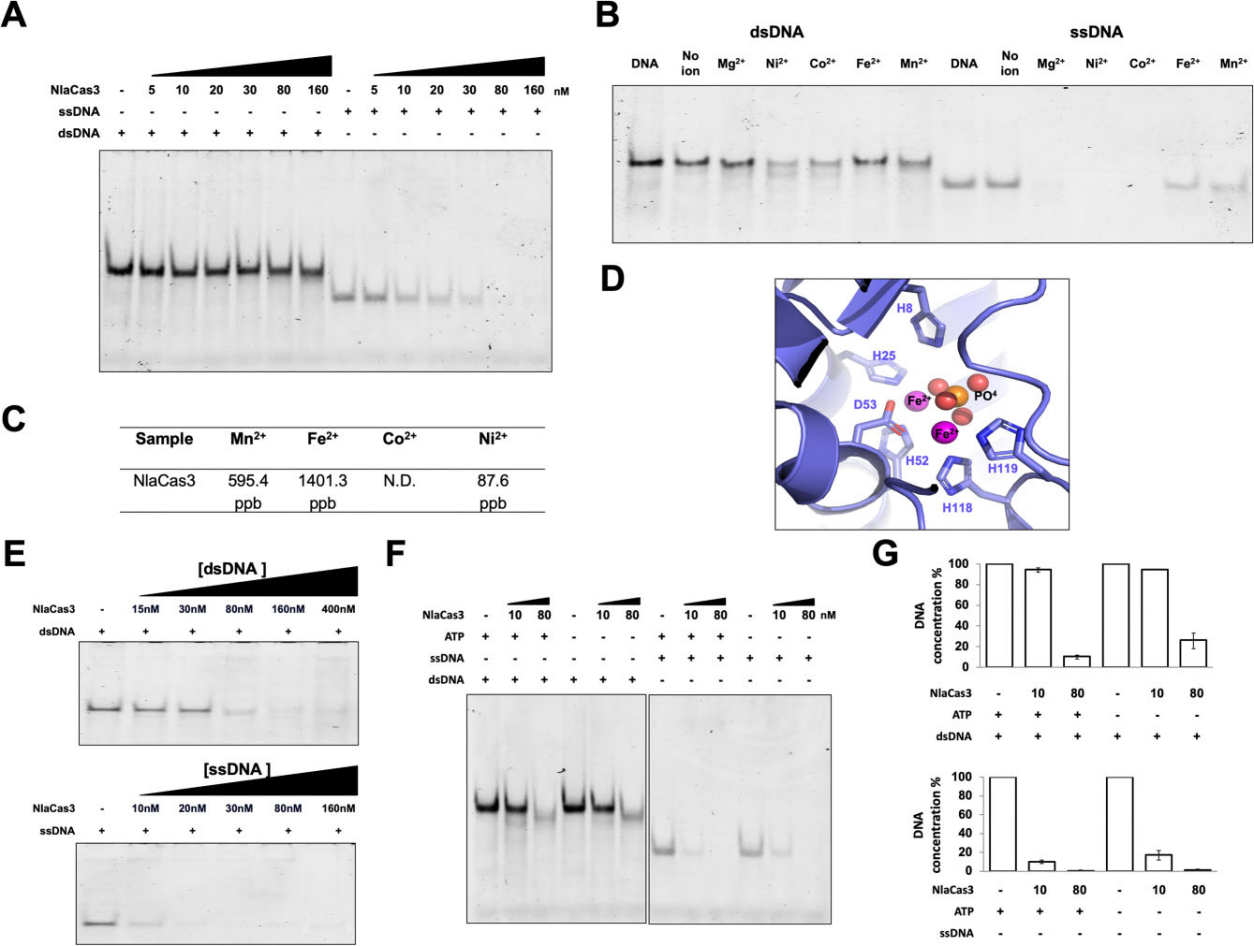
NlaCas3 can cleave both ssDNA and dsDNA in a metal ion-dependent manner. (**A**) Degradation of ssDNA and dsDNA by NlaCas3. NlaCas3 was incubated with 30 nM ssDNA or dsDNA at 37°C for 2 h in the presence of 6 mM MgCl_2_. In the enzyme reaction, + and – indicate added and not added, respectively. (**B**) Metal ion-dependent cleavage of ssDNA and dsDNA by NlaCas3. NlaCas3 (80 nM) was incubated with 30 nM dsDNA at 37°C for 2 h in the presence of different metal ions. For analyzing ssDNA cleavage, 20 nM NlaCas3 was incubated with 30 nM ssDNA at 37°C for 2 h in the presence of different metal ions. In both cases, 8 mM of ATP was added to the reaction mixture. (**C**) Table showing the metal ion concentration in NlaCas3 analyzed by ICP–MS. N.D., not determined. (**D**) Magnified cartoon of the Fe^2+^-binding site of NlaCas3. Residues that coordinate with the metal ion are labelled. (**E**) Optimized cleavage condition for NlaCas3. NlaCas3 was incubated with 30 nM ssDNA or dsDNA at 37°C for 2 h in the presence of 6 mM MgCl_2_, 0.4 mM NiCl_2_, and 8 mM ATP. In the enzyme reaction, + and – indicate added and not added, respectively. (**F**) Analysis of the effect of ATP on NlaCas3 activity. NlaCas3 [10 nM (low) and 80 nM (high)] was incubated with 30 nM ssDNA or dsDNA at 37°C for 2 h in the presence of 6 mM MgCl_2_ and 0.4 mM NiCl_2_ in the presence or absence of 8 mM ATP. (**G**) Quantitative histogram of Cas3 activity according to (**F**). Substrate DNA concentration provided for Cas3 activity analysis was considered to be 100% of DNA concentration. Data are presented as the mean ± standard deviation from three independent experiments.

Most of the characterized Cas3 enzymes cleave ssDNA (34,35). We initially investigated the cleavage efficiency of Type I-C NlaCas3 on both ssDNA and dsDNA in the presence of Mg^2+^, a crucial cofactor for nucleolytic activity. NlaCas3, in the presence of Mg^2+^, could not cleave dsDNA, whereas ssDNA was cleaved in a concentration-dependent manner of the enzyme (Figure 2A).

Several Cas proteins exhibit activity with metal ions serving as crucial cofactors, and ions of various types influence their activities (20–22). Therefore, we investigated the ion preference of NlaCas3. For this analysis, we tested the cleavage capability and efficiency of 20–80 nM NlaCas3 for both dsDNA and ssDNA in the presence of Mg^2+^, Ni^2+^, Co^2+^, Fe^2+^ and Mn^2+^. In the absence of ions, dsDNA could not be cleaved by NlaCas3, and Ni^2+^ or Co^2+^ facilitated better cleavage than did the other ions. Similarly, no ssDNA cleavage was observed in the absence of ions, whereas ssDNA was cleaved in the presence of Mg^2+^, Ni^2+^ or Co^2+^ (Figure 2B).

Two prominent round-shaped electron densities, characteristic of metal-ion density, were observed around the active site of HD nuclease of NlaCas3 (Figure 1G). This metal ion density was coordinated by O and N atoms from the side chains of residues H8, H25, H52, D53, H118 and H119 (Figure 1G). The distance between the metal ion and coordinated oxygen and nitrogen atoms from the side chain was <3.5 Å. Using ICP–MS, we identified the metal ion as Fe^2+^ or Mn^2+^ with a concentration of 1401.3 or 595.4 ppb, respectively (Figure 2C). Therefore, Fe^2+^ and Mn^2+^ were bound to NlaCas3. Additionally, ICP–MS results indicating a 2:1 ratio of Fe^2+^ to Mn^2+^ binding indicated that the metal ions in the active site of HD nuclease domain corresponded to two Fe^2+^ ions (Figure 2D). We did not find any additional metal density fit for Mn^2+^. Based on the ion-binding studies and experimental results of NlaCas3 activity, we systematically investigated how Cas3 activity varies in a concentration-dependent manner in environments containing Mg^2+^ or Ni^2+^, the most effective ions for Cas3 activity. In the presence of 6 mM Mg^2+^ and 0.4 mM Ni^2+^, approximately 80 nM Cas3 was sufficient in completely cleaving 200 nM dsDNA, while approximately 10–20 nM Cas3 fully cleaved 200 nM ssDNA (Figure 2E).

Cas3 contains a RecA-like helicase domain, allowing it to unwind target DNA molecules, which relies on ATP for its functionality (34). Therefore, we assessed the cleavage efficiency of NlaCas3 on dsDNA and ssDNA in the presence or absence of ATP and in the presences of Mg^2+^ or Ni^2+^ (Figure 2B and E). ssDNA was efficiently cleaved even at low concentration of NlaCas3 (10 nM), regardless of the presence of ATP, whereas for dsDNA, minimal cleavage was observed only at high enzyme concentration (80 nM) (Figure 2F and G). Interestingly, at high NlaCas3 concentration, dsDNA cleavage occurred in the absence of ATP (Figure 2F and G). However, the extent of cleavage was weaker than that in the presence of ATP. Therefore, in the presence of Mg^2+^, Ni^2+^ or Co^2+^, NlaCas3 can efficiently cleave ssDNA even at low concentrations, independent of ATP, and exhibit minimal cleavage of dsDNA at high concentrations in the presence of Ni^2+^ and Mg^2+^ ions, irrespective of the presence of ATP. However, ATP somewhat influenced Cas3 activity on dsDNA.

### Type I-C NlaCas3 significantly differs in structure and amino acid sequence from those of other types Cas3 from different species

**Figure 3.**
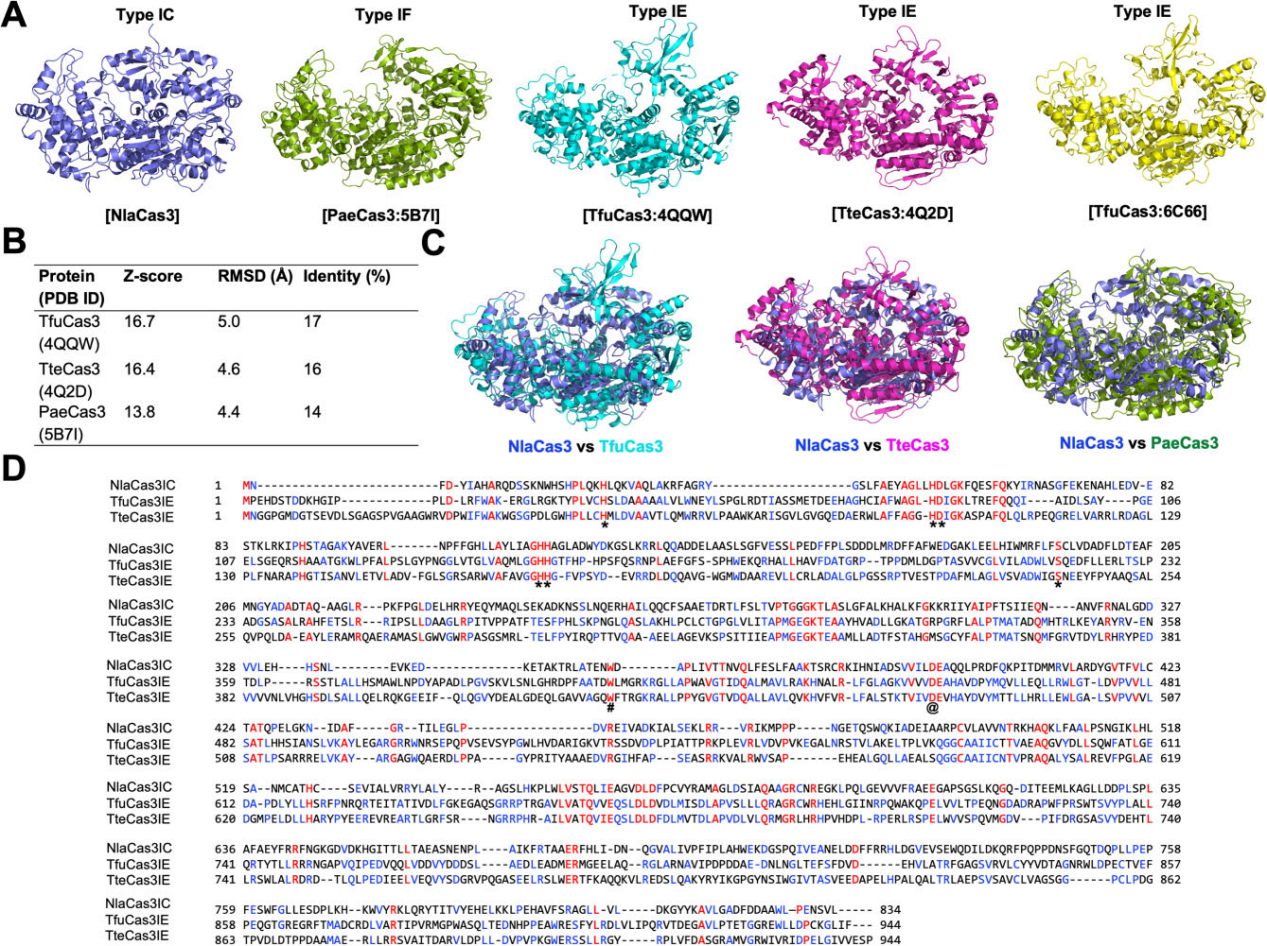
The structure and amino acid sequence of NlaCas3 significantly differ from those of other types of Cas3. (**A**) Structures of various types of Cas3. (**B**) Table summarizing the result of structural similarity search using DALI server. (**C**) Pairwise structural comparison of NlaCas3 with different types of Cas3 by structural superposition. (**D**) Sequence alignment of Type I-C NlaCas3 with Type I-E Cas3 using Geneious Prime v.2024 0.3 (https://www.geneious.com). Identical amino acid residues are highlighted in red, and similar amino acids are shown in blue. Amino acid residues marked with * were identified as crucial for the HD nuclease activity of TFuCas3, and those marked with @ were found to be important for the ATPase activity. # indicated the residue critical for the helicase activity of TteCas3.

Structural studies of standalone Cas3 have primarily focused on Cas3 belonging to Type I-E, particularly those from *Thermobifida fusca* and *Thermobaculum terrenum* (20,22,23). Recently, structural investigations of standalone Type I-F Cas3 from *Pseudomonas aeruginosa* have been performed (21). Although the structures of several types of standalone Cas3, including Type I-E and Type I-F, have been revealed, the structure of standalone Type I-C Cas3 remains unexplored (Figure 3A). Therefore, the structure of Type I-C NlaCas3 was compared with those of other types of NlaCas3 utilizing the DALI server for searching structural homologs (36). The DALI server provided three structurally similar proteins: Cas3 from *T. fusca* (PDB ID: 4QQW, hereafter referred to as TfuCas3), Cas3 from *T. terrenum* (PDB ID: 4Q2D, hereafter referred to as TteCas3), and Cas3 from *P. aeruginosa* (PDB ID: 5B7I, hereafter referred to as PaeCas3) (Figure 3B). Among them, TfuCas3 exhibited the highest similarity, having a *Z*-score of 16.7, root mean square deviation (RMSD) of 5.0 Å, and sequence identity of 17%, followed by TteCas3, while PaeCas3 showed the lowest similarity. These results suggest that NlaCas3 structure is more closely related to Type I-E structures than to that of Type I-F Cas3. Overall, DALI analysis indicated significant differences in both structure and amino acid sequence between Type I-C NlaCas3 and other types of Cas3 proteins. Pairwise structural superposition analysis further confirmed that NlaCas3 structure did not align well with those of other Cas3 proteins (Figure 3C).

Despite the low sequence identity of amino acids, residues H37, H83, D84, H149, H150 and S219 of TfuCas3, which were previously identified as crucial for HD nuclease function (20), were well-conserved as H8, H52, H53, H118, H119 and S192, respectively, in NlaCas3 (Figure 3D). Additionally, D451 of TfuCas3, which is important for ATPase activity (20), was also well conserved as D392 in NlaCas3 (Figure 3D). Lastly, we observed that the residue W432, known to be important for helicase activity in TteCas3 (22), was also well-conserved in NlaCas3 as W354 (Figure 3D). These findings suggest that despite differences in structures and sequences among different Cas3 protein from various species, the mechanisms of their operation are likely to be similar.

### Identification of residues critical for the nuclease activity of NlaCas3

**Figure 4.**
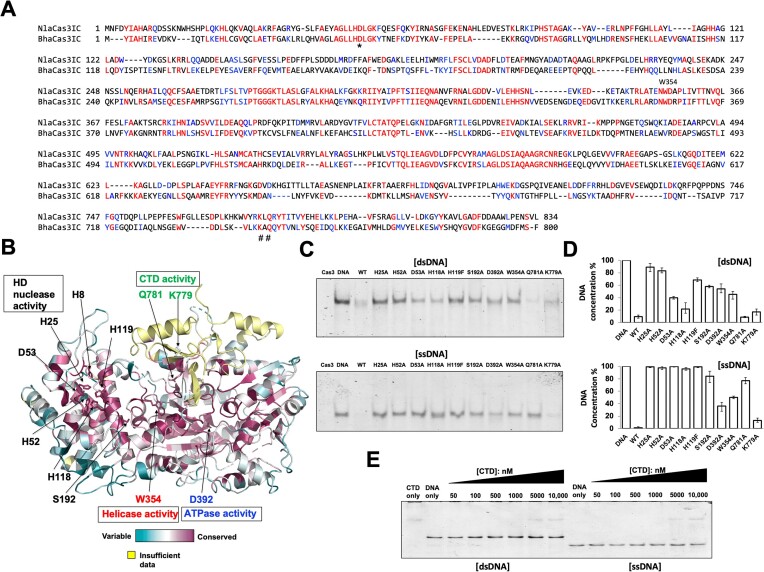
Identification of residues critical for the nuclease activity of NlaCas3. (**A**) Sequence alignment of Type I-C NlaCas3 with BhaCas3. Identical amino acid residues are highlighted in red, and similar amino acids are shown in blue. Amino acid residues marked with * were identified as crucial for the HD nuclease activity of BhaCas3, and those marked with # were found to be important for the role of CTD. (**B**) Cartoon representation illustrating the conservation of amino acids across different Cas3 family members, as analyzed using ConSurf. Residues speculated as important for each function of NlaCas3 are labeled. (**C**) Degradation of dsDNA and ssDNA by various NlaCas3 mutants. NlaCas3 or various NlaCas3 mutants (80 nM) were incubated with 30 nM dsDNA at 37°C for 2 h in the presence of 6 mM MgCl_2_, 0.4 mM NiCl_2_, and 8 mM ATP. For ssDNA cleavage, 20 nM of NlaCas3 or various NlaCas3 mutants were incubated with 30 nM ssDNA at 37°C for 2 h in the presence of 6 mM MgCl_2_, 0.4 mM NiCl_2_, and 8 mM ATP. (**D**) Quantitative histogram of Cas3 mutants activity according to (C). Substrate DNA concentration provided for Cas3 mutants activity analysis was considered to be 100% of DNA concentration. Data are presented as the mean ± standard deviation from three independent experiments. (**E**) EMSA with CTD of NlaCas3 using dsDNA or ssDNA in a various different concentration of protein. Non-denaturing acrylamide gels stained with SYBR Gold are shown.

Among Type I-C Cas3 proteins, extensive biochemical activity studies have been conducted without structural information on Cas3 from *Bacillus halodurans* (hereafter referred to as BhaCas3) (35). In this study, D48 plays a crucial role in the HD nuclease activity of BhaCas3. Additionally, K743 and Q781 in the CTD were found to be important for CTD function, and mutations at these sites resulted in the loss of nuclease activity in BhaCas3. When aligning the amino acid sequences of NlaCas3 with BhaCas3, we found that the sequence identity was only approximately 38% despite being the same type. However, the residues, including D48, K743 and Q745, which are critical for BhaCas3 activity were well conserved as D53, K779 and Q781, respectively, in NlaCas3 (Figure 4A). Interestingly, the two residues, K779 and Q781, deemed important for CTD function in Type I-C Cas3 were not conserved in Type I-E Cas3, such as TfuCas3 (Figure 3D).

Based on previous studies on Cas3 activity and ConSurf analysis identifying highly conserved amino acids that are likely crucial for the function of Cas3 (37), we selected amino acids that were predicted to play key roles in NlaCas3 activity (Figure 4B) and conducted mutation studies to validate our hypotheses. We selected seven residues in the active site predicted to be crucial for HD nuclease activity and mutated six of them (H25, H52, D53, H118, H119 and S192) to alanine. Additionally, W354 and D392, predicted to be important for the helicase and ATPase activities, respectively, were mutated to alanine. Based on the findings of the BhaCas3 study, we further mutated K779 and Q781, which are predicted to be crucial for CTD function, to alanine. All mutant proteins were purified and subjected to biochemical activity assays for validating our hypothesis. As expected, mutations in all active-site residues, including H25A, H52A, D53A, H118A, H119A and S192A, resulted in a loss of activity of HD nuclease against dsDNA and ssDNA (Figure 4C and D). Nevertheless, we observed that dsDNA cleavage seems only partially impaired for D53A and H118A. As anticipated, mutations at W354 and D392 resulted in a loss of activity against dsDNA. Unexpectedly, those mutants, W354 and D392, activities were also affected on ssDNA (Figure 4C and D). Interestingly, mutations at K779 and Q781 did not affect NlaCas3 activity on dsDNA (Figure 4C). However, while K779A mutation still exhibited activity on ssDNA, Q781A mutation resulted in loss of activity on ssDNA (Figure 4C). Therefore, Q781 plays a crucial role in the CTD function of NlaCas3 on ssDNA. Although the precise function of the Cas3 CTD has not yet been elucidated, earlier studies on Type I-E Cas3 suggest that the CTD functions as a ‘substrate filter’ and transiently dissociates to allow non-target strand ssDNA recruitment into the helicase domain of Cas3, thereby positively affecting Cascade binding affinity without binding to DNA (20). To determine whether the CTD of NlaCas3 binds to DNA to facilitate the nuclease function of Cas3, we conducted EMSA experiments. The results showed that the CTD domain alone did not exhibit any binding (Figure 4E). These results suggest that the CTD of NlaCas3 likely acts as a filter to facilitate the access of the DNA substrate to the active site, rather than directly binding to the substrate for nuclease function of Cas3. The precise role of the CTD will need to be investigated in future studies.

### NlaCas3 undergoes structural changes in the anchor loop for binding to the Cascade complex and gate loops for accommodating DNA substrates

**Figure 5.**
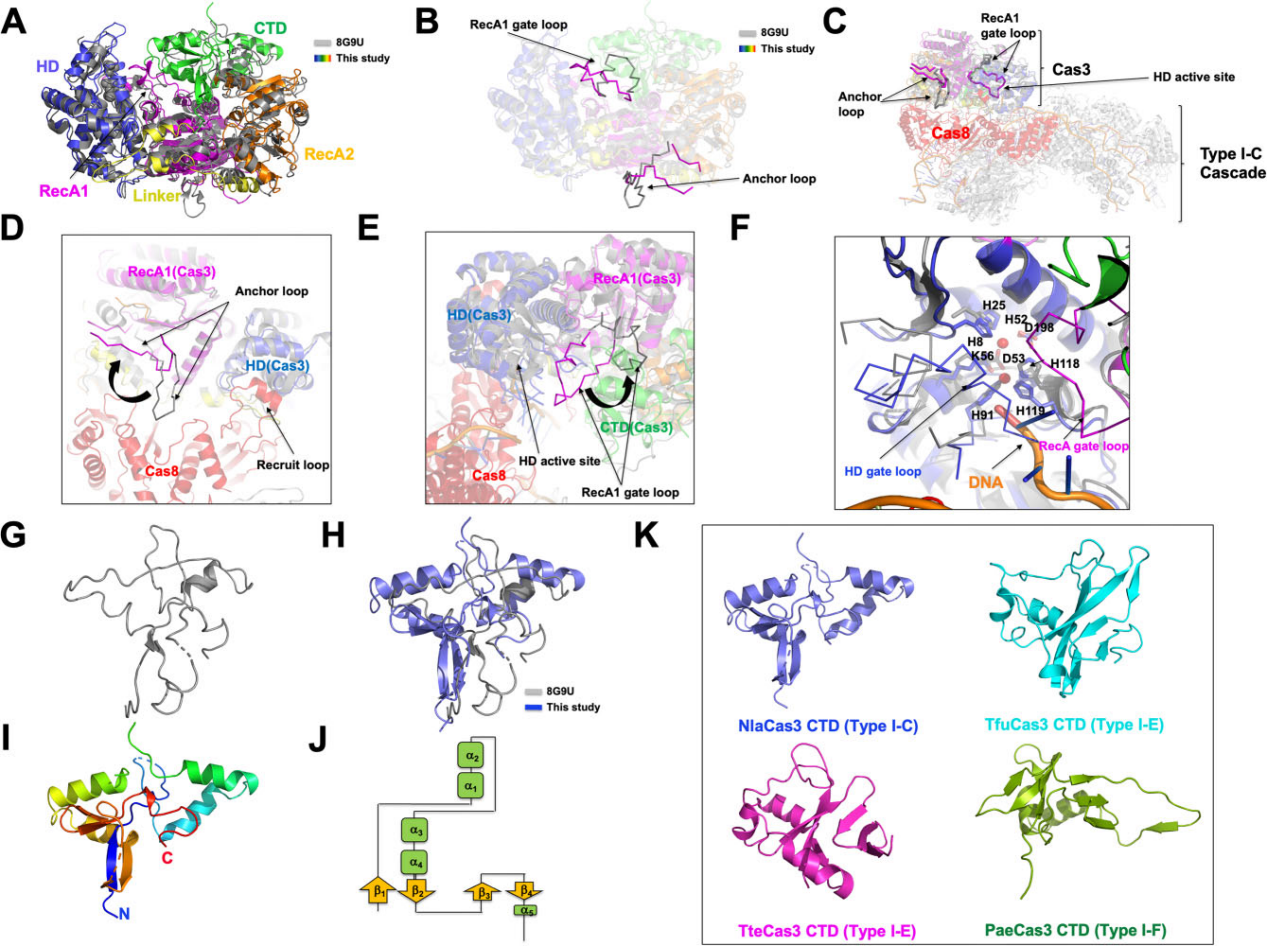
Comparative structural analysis of inactive and active states of NlaCas3. (**A**) Structural comparison between NlaCas3 in the Cascade complex (PDB ID: 8G9U and gray color) and our isolated NlaCas3 (colorful model) through superposition. (**B**) The anchor and RecA1 gate loops, marked with black arrows, exhibit the most pronounced structural changes between these two structures. The color scheme corresponds to that of panel A. (**C**) Figure illustrating the positions of two loops exhibiting the most pronounced structural changes in the Cascade–Cas3 complex. The two loops are labelled, and Cas8 of Cascade, which directly interacts with Cas3, is highlighted in red. To generate this figure, we aligned our isolated NlaCas3 structure with the Cascade–Cas3 complex structure (PDB ID: 8G9U). (**D**) An enlarged view of the anchor loop region from panel C, with structural shifts indicated by rotating arrows. The recruit loop of Cas8, which is involved in binding to Cas3, is also highlighted in the diagram. (**E**) An enlarged view of the RecA1 gate loop region from panel C, with structural shifts indicated by rotating arrows. (**F**) A close-up view of the active site of HD nuclease from panel C. The two loops exhibiting the most significant structural changes before and after complex formation between Cascade and Cas3 at the active site are labelled as the HD and RecA1 gate loops, respectively. (**G**) The structure of CTD derived from Cas3, forming part of the Cascade complex (PDB ID: 8G9U). (**H**) Comparison between the CTD structure of our NlaCas3 and NlaCas3 from the Cascade complex structure by superposition. (**I**) The overall structure of NlaCas3 CTD derived from our current structure. The color of the chain from the N- to C-terminus gradually moves through the spectrum from blue to red. (**J**) Topology representation of the CTD of NlaCas3. (**K**) Structural diversity of CTDs from various Cas3 proteins of different species.

The structure of Type I-C Cascade complex, including Cas3, has recently been published (PDB ID: 8G9U) (24). As this complex represents Cas3 in its active state, we assumed that comparing its structure with the current structure of NlaCas3, representing a tentative inactive state, would enable a comparative analysis between the active and inactive forms of NlaCas3. To compare the two structures, we superimposed the structure of Cas3 in complex with Cascade (hereafter referred to as Cas3^Cascade^) on the structure of our isolated NlaCas3. Although the overall appearance of the two structures was very similar, with an RMSD of approximately 1.5 Å, some regions exhibited completely different structures (Figure 5A). The most structurally distinct region was the anchor loop, which was one of the two regions (anchor and recruit loops) described in the structure of Cas3–Cascade complex as being used by Cas3 for strongly binding to Cascade (Figure 5B). Another region showing significant structural differences was the loop emerging from the RecA1-like helicase domain, which was directed towards the active site of HD nuclease (Figure 5B). This region is referred to as the RecA1 gate loop. After observing these structural differences, we superimposed the Cas3-alone structure on Cas3^Cascade^ to determine the precise location of the two distinct loops within the complex structure. The anchor loop of Cas3 played a crucial role in binding to Cas8 within the Cascade complex (Figure 5C). However, when Cas3 was used alone, the anchor loop significantly moved in the direction opposite to that of Cas8, indicating no impact on binding with Cas8 (Figure 5C and D). Therefore, when Cas3 is present alone, the anchor loop may exist elsewhere; however, upon binding with Cascade for activation, it undergoes structural changes to facilitate binding with Cas8 in the Cascade complex. These structural changes play a crucial role in determining how Cas3 anchors to Cas8, thereby highlighting the importance of the anchor loop in this process. Additionally, the RecA1 gate loop moved away from the active site of HD nuclease upon binding, whereas in our structure without Cascade binding, the loop was oriented towards the active site of HD nuclease (Figure 5C and E). The RecA1 gate loop, which blocks the active site of HD nuclease, underwent structural changes for moving away from the active site, thereby allowing access for DNA binding (Figure 5F). Notably, we identified an additional loop in our Cas3 structure, which could potentially lead to steric hindrance upon binding of DNA, as it obstructs the active site (Figure 5F). We termed this loop the HD gate loop, recognizing that it originated from HD nuclease. The HD gate loop in Cascade–Cas3 complex was structurally undefined in active Cas3 (Figure 5F). This is probably because it is a highly flexible loop that may undergo significant structural changes to accommodate substrate binding. Therefore, determining its structure in active Cas3 was challenging owing to the difficulty in defining its conformation amid these structural dynamics.

When comparing the two structures, besides the two loops, the most prominent difference was noticed in the overall structure of the CTD. In the actual structure of Cascade–Cas3 complex, the CTD structure of active Cas3 was mostly incomplete and consisted mainly of loops (Figure 5G and H). This suggests that the structure of this region remained unresolved because of its low resolution. However, in our structure of NlaCas3 alone, the CTD structure was well defined. The CTD structure of NlaCas3 exhibited a globular fold comprising five α-helices and four β-strands (Figure 5I and J). The CTD structure represented the first elucidated CTD structure of Type I-C Cas3, prompting us to compare it with previously determined CTD structures of Cas3 from other types. We observed significant variations in CTD structures across different types (Figure 5K). Interestingly, even within the same type, considerable diversity was observed in CTD structures.

## Discussion

Cas3 is the main nuclease in the Class I CRISPR-Cas system, and plays a crucial role in forming a full Cascade complex for recognizing and cleaving target DNA. The mechanism of action of Cas3 has been extensively studied, particularly focusing on the structure of Type I-E Cas3 (20,23). However, the molecular mechanism of Type I-C Cas3 remains unclear owing to the lack of structural studies. *N. lactamica* contains a Type I-C CRISPR-Cas3 system. In this study, we elucidated the structure of NlaCas3. Our structural study revealed that, like other types of Cas3, NlaCas3 is distinctly composed of three major domains including the HD nuclease domain, RecA-like helicase domain, and CTD. Additionally, it contains a linker region that connects the RecA-like helicase domain to the CTD.

The active site of the HD nuclease domain in NlaCas3 contains two Fe^2+^ ions that play crucial roles in functioning and efficiency of Cas3. Type I-E Cas3, TfuCas3, also contains two Fe^2+^ ions bound to its active site (20,23). In contrast, the Type I-E Cas3, TteCas3, has three Ni^2+^ ions (22), and the Type I-F Cas3, PaeCas3, has Ca^2+^ ions (21). These specific metal ion bindings seem to be critical for regulating Cas3 activity. For instance, TfuCas3 shows optimal activity on ssDNA in the presence of Mg^2+^, Mn^2+^, and Co^2+^; however, its activity is inhibited by Ni^2+^, Cu^2+^ and Fe^2+^ (20). TteCas3 exhibits the highest activity with Mn^2+^ and Ni^2+^ (22). PaeCas3 shows the highest activity with Mn^2+^ and Fe^2+^, moderate activity with Ni^2+^ and Mg^2+^, and little activity with Zn^2+^ (21). For the newly studied Type I-C Cas3, NlaCas3, specific metal ions, such as Ni^2+^ and Co^2+^, facilitated activity on both ssDNA and dsDNA, while Mg^2+^ also induced considerable activity on ssDNA. However, Fe^2+^ and Mn^2+^ did not induce such activity. These findings suggest that various metal ions can bind to the active site of HD nuclease domain in Cas3, and that the bound ion determines the nuclease activity of Cas3. Different types of Cas3 exhibit distinct preferences for metal-ion binding and activity. For NlaCas3, the inactive state appears to be associated with Fe^2+^, and activation may occur in the presence of Mg^2+^, Ni^2+^ and Co^2+^.

ATP is a crucial factor for helicase activity of Cas3. Cas3 uses the energy derived from ATP hydrolysis within its RecA-like helicase domain to unwind dsDNA into ssDNA strands that are then cleaved by the HD nuclease domain of Cas3. ATP is essential for Cas3 function. NlaCas3 exhibited activity on both ssDNA and dsDNA in the presence of Mg^2+^ or Ni^2+^, regardless of the presence of ATP. However, Cas3 activity on dsDNA was higher in the presence of ATP. In summary, NlaCas3 exhibited minimal activity on dsDNA, even without ATP, under specific ion conditions; however, its activity was enhanced in the presence of ATP, indicating that ATP strengthens its activity under conditions where specific ions (Mg^2+^ or Ni^2+^) are present. For Cas3, it is well-known as an ssDNA cutter that cleaves the ssDNA created by the bubble loop formed by the Cascade (19,23). However, in our current study and other research, particularly with other type I-C Cas3, it has been observed that it can also cleave dsDNA under certain conditions (35). The fact that Cas3 can cut dsDNA is a very dangerous phenomenon, as it can target the host genome and cause self-damage. In our current study, it was observed that NlaCas3 cuts dsDNA only at very high concentrations and in the presence of specific ions, such as Ni^2+^ and Co^2+^. This action of NlaCas3 might be an artificial result that occurs at concentrations not typically found in actual cells, or it could represent a real cellular mechanism where ion regulation controls Cas3 activity when high concentrations are present. Further research is needed to fully understand the dsDNA cutting phenomenon of Cas3.

Cas3 proteins exhibited low sequence identity of amino acids, ranging from 14–17% across different types, resulting in structurally diverse forms. Even within the same Type I-C, for instance, BhaCas3 shared only approximately 38% sequence identity of amino acids. This highlights considerable diversity in the evolution of Cas3 structures and forms. Despite this diversity, the essential amino acids crucial for Cas3 function are well conserved. The key residues crucial for the HD nuclease activity of NlaCas3 were found to be H8, H25, H52, D53, H118, H119 and S192. Experimental evidence validated the importance of these residues for NlaCas3 activity on dsDNA and ssDNA. Therefore, although Cas3 proteins exhibit structural diversity, their mechanisms of action may be similar. Furthermore, we identified W354 and D392 in NlaCas3 as important residues for helicase and ATPase activities, respectively. Mutations at these sites affected Cas3 activity, indicating the significance of helicase and ATPase functions in nuclease activity of NlaCas3. However, D392A mutation retained some level of NlaCas3 activity, suggesting that ATPase function minimally influences the nuclease activity of NlaCas3. Interestingly, two residues known to play important roles in the function of the Type I-C Cas3 CTD, K779 and Q781, had distinct roles in NlaCas3. Although these two residues were not crucial for cleavage of dsDNA, Q781 was important for action on ssDNA, as demonstrated by our mutation experiments. These findings suggest that the CTD influences the nuclease activity of NlaCas3 on ssDNA, and that its action varies depending on whether it interacts with ssDNA or dsDNA. Exploration of the precise mechanisms of action of the CTD on ssDNA and dsDNA could be an intriguing avenue for future research.

The Cryo-EM structure of Type I-C Cascade system consisting of NlaCascade and NlaCas3 has recently been elucidated (24). The authors have structurally elucidated how Cas3 binds to Cascade and the mechanism by which Cas3 recognizes substrates after binding to Cascade. They demonstrated that Cas3 and Cascade utilize the anchor loop from Cas3 and recruit loop from Cas8 in the Cascade complex for binding to each other. When we compared our standalone NlaCas3 structure with that of NlaCas3 in complex with Cascade, the anchor loop of NlaCas3, which is utilized for binding with Cas8 in the complex, underwent significant structural changes when NlaCas3 was present alone and positioned in a completely opposite direction. In contrast, no change in the structure of NlaCas3 that interacts with the recruit loop of Cas8 was noticed. Therefore, NlaCas3 undergoes structural changes in its anchor loop upon binding to Cascade, allowing it to be inserted into the binding site of Cas8. This mechanism suggests that Cascade recognizes and binds Cas3 using its recruit loop, and Cas3, akin to filling a clip with its anchor loop, establishes a relatively strong interaction to secure Cas3 in the optimal position for cleaving target DNA. This prediction implies that the anchor loop plays a crucial role in anchoring Cas3 to the target DNA for efficient cleavage, once recruited by Cascade. When comparing our standalone NlaCas3 structure in its presumed inactive form to the structure of Cas3 bound to the actual substrate DNA within the Cascade complex, we observed that the HD gate loop from the HD nuclease domain and RecA1 gate loop from the RecA1 domain in our NlaCas3 structure blocked the active site of HD nuclease. However, in NlaCas3, within the active Cascade complex, these loops are found to be open. This structural difference indicated that substrate access to the active site is regulated by these two loops. We hypothesized that this structural alteration, which regulates substrate access, is a highly sophisticated process that is crucial for precise DNA recognition and cleavage.

Mg^2+^ was identified within active NlaCas3 in the Cascade complex. This suggests that NlaCas3 adopts an active form when bound to Mg^2+^, as Mg^2+^ ions enhance its activity on ssDNA. Conversely, in our isolated NlaCas3 structure, Fe^2+^ ions, which inhibit NlaCas3 activity, were detected, indicating an inactive form. This supports our hypothesis that the presence of Fe^2+^ corresponds to the inactive state of NlaCas3. We observed structural changes between the inactive form we described and the active form within the Cascade complex in several regions, including anchor loop, HD gate loop, and RecA1 gate loop. However, it remains to be determined whether these structural changes are due to the different ions or the interaction between Cas3 and Cas8 in the Cascade complex. Our current study does not provide this distinction, which will need to be addressed in future research.

The structure of CTD of active NlaCas3 in the Cascade complex has been poorly modeled, probably because of unclear electron density in this region, indicating its undetermined structure. Conversely, in its isolated inactive state, our structural analysis provided relatively clear insights into the CTD structure of NlaCas3. This suggests that the CTD operates dynamically during Cas3 activation, as it is connected to the preceding RecA-like helicase domain via a long linker domain, indicating potential dynamic movements of the CTD. Interestingly, the structures of CTDs significantly varied among different types of Cas3 proteins, and even within the same type, different structures were observed. This wide diversity in amino acid sequences and structures among CTDs raises questions regarding how diverse CTDs perform the same function in Cas3. These phenomena, along with the changes in mechanism of activation owing to different metals, and real-time dynamics of various loops during the actual Cas3 activation process, warrant further exploration.

## Supplementary Material

gkae723_Supplemental_File

## Data Availability

The coordinate and structure factor have been deposited to the Research Collaboratory for Structural Bioinformatics (RCSB) Protein Data Bank (PDB) under the PDB code 8ZNS.
