# Supplementary material for: Structural basis of Cas3 activation in type I-C CRISPR-Cas system
Source: Nucleic Acids Res. 2024 Aug 24;52(17):10563–74. doi: 10.1093/nar/gkae723 (PMC11417383; doi:10.1093/nar/gkae723)
Supplement: gkae723_Supplemental_File [file gkae723_supplemental_file.docx]

**Supplementary Table1.** Oligonucleotides used in this study.

| Name | Sequence (5’→3’) |
| --- | --- |
| H25A(Mut)- sense | GCGGGGCTTTTGGCCGATTTGGGGAAATT |
| H25A(Mut)- antisense | AATTTCCCCAAATCGGCCAAAAGCCCCGC |
| H52A(Mut)- sense | TATCCCCTGCAAAAAGCTCTACAAAAAGTCGC |
| H52A(Mut)- antisense | GCGACTTTTTGTAGAGCTTTTTGCAGGGGATA |
| D53A(Mut)- sense | GGGGCTTTTGCACGCTTTGGGGAAATTTG |
| D53A(Mut)- antisense | CAAATTTCCCCAAAGCGTGCAAAAGCCCC |
| H118A(Mut)- sense | ATTTGATTGCCGGGGCTCATGCTGGGCTGGCAG |
| H118A(Mut)- antisense | CTGCCAGCCCAGCATGAGCCCCGGCAATCAAAT |
| H119F(Mut)- sense | TTGCCGGGCATTTTGCTGGGCTGG |
| H119F(Mut)- antisense | CCAGCCCAGCAAAATGCCCGGCAA |
| S192A(Mut)-sense | GCGTTTTCTCTTTGCCTGCTTGGTGGATGC |
| S192A(Mut)-antisense | GCATCCACCAAGCAGGCAAAGAGAAAACGC |
| W354A(Mut)- sense | CGTGGTGATTTTGGCTGAAGCCCAGCTTC |
| W354A(Mut)- antisense | GAAGCTGGGCTTCAGCCAAAATCACCACG |
| D392A(Mut)- sense | CTACGGAAAATGCGGACGCGCCGC |
| D392A(Mut)- antisense | GCGGCGCGTCCGCATTTTCCGTAG |
| K779A(Mut)- sense | ATGGGTTTACCGCAAGCTGCAACGCTAC |
| K779A(Mut)- antisense | GTAGCGTTGCAGCTTGCGGTAAACCCAT |
| Q781A(Mut)- sense | TACCGCAAGCTGGCACGCTACACGATTAC |
| Q781A(Mut)- antisense | GTAATCGTGTAGCGTGCCAGCTTGCGGTA |
| Cas3 Target dsDNA  sense | AGGGCGAGGGCGATGCCACCTACGGCAAGCTGACCCTGAAGT |
| Cas3 Target dsDNA  Anti-sense | ACTTCAGGGTCAGCTTGCCGTAGGTGGCATCGCCCTCGCCCT |
| Cas3 Ttarget ssDNA | ACTTCAGGGTCAGCTTGCCGTAGGTGGCATCGCCCTCGCCCT |
